# Influential Factors and Personalized Prediction Model of Acute Pain Trajectories after Surgery for Renal Cell Carcinoma

**DOI:** 10.3390/jpm12030360

**Published:** 2022-02-26

**Authors:** Hsin-Jung Tsai, Wen-Kuei Chang, Fang-Yu Yen, Shih-Pin Lin, Tzu-Ping Lin, Kuang-Yi Chang

**Affiliations:** 1 Department of Anesthesiology, Taipei Veterans General Hospital, Taipei 112201, Taiwan; hj.hjtsai@gmail.com (H.-J.T.); wkchang@vghtpe.gov.tw (W.-K.C.); aslam.lin@gmail.com (S.-P.L.); 2 School of Medicine, National Yang Ming Chiao Tung University, Taipei 112304, Taiwan; fangyuyen@gmail.com (F.-Y.Y.); tplin@vghtpe.gov.tw (T.-P.L.); 3 Department of Anesthesiology, Taoyuan Armed Forces General Hospital, Taoyuan City 32551, Taiwan; 4 Department of Urology, Taipei Veterans General Hospital, Taipei 112201, Taiwan

**Keywords:** epidural analgesia, latent curve model, pain trajectory, patient-controlled analgesia, renal cell carcinoma

## Abstract

Background: Renal cell carcinoma (RCC) is the most common neoplasm in kidneys, and surgical resection remains the mainstay treatment. Few studies have investigated how the postoperative pain changes over time and what has affected its trajectory. This study aimed to characterize the variations in postoperative pain over time and investigate associated factors after RCC surgery. Methods: This retrospective study was conducted in a single medical center in Taiwan, where maximal pain scores in a numeric rating scale were recorded daily in the first five postoperative days (PODs) after RCC surgery. Latent curve models were developed, using two latent variables, intercept and slope, which represented the baseline pain and rate of pain resolution. These models explain the variations in postoperative pain scores over time. A predictive model for postoperative pain trajectories was also constructed. Results: There were 861 patients with 3850 pain observations included in the analysis. Latent curve analysis identified that female patients and those with advanced cancer (stage III and IV) tended to have increased baseline pain scores (*p* = 0.028 and 0.012, respectively). Furthermore, patients over 60 years, without PCA use (both *p* < 0.001), and with more surgical blood loss (*p* = 0.001) tended to have slower pain resolution. The final predictive model fit the collected data acceptably (RMSEA = 0.06, CFI = 0.95). Conclusion: Latent curve analysis identified influential factors of acute pain trajectories after RCC surgery. This study may also help elucidate the complex relationships between the variations in pain intensity over time and their determinants, and guide personalized pain management after surgery for RCC.

## 1. Introduction

Renal cell carcinoma (RCC) accounts for more than 90% of cancers in the kidney [1]. Surgical excision through radical or partial nephrectomy remains the standard curative treatment [1]. However, patients undergoing open nephrectomy experience relatively severe postoperative pain due to the long incision, with or without rib resection [2]. Postoperative pain management in these patients is a challenging subject. Poor pain management after surgery causes delayed recovery, immobility, activation of stress responses, and increased incidence of cardiac and pulmonary complications [3,4,5,6]. Furthermore, inappropriate treatment of acute postoperative pain tends to lead to the development of chronic postsurgical pain [7,8,9,10].

Many factors are involved in the etiology of postoperative pain, from biological, psychosocial, to functional elements [11,12]. In addition, a patient’s age, sex, body weight, and the type of surgery are found to predict the development of severe postoperative pain [13,14]. In order to analyze the complex interactions between management and symptoms, pain trajectory analysis may be a better assessment tool than individual pain measurements, since the intensity and characteristics of pain vary over time [15,16]. Postoperative pain trajectories are associated with various clinical outcomes, such as chronic postsurgical pain [17,18,19,20], postoperative readmission [21], functional disability [22], and the outcome of cancer surgery [23]. In addition, patients classified in a higher pain group than the average pain trajectory had longer durations of pain and opioid use [24]. Accordingly, analyzing the time course of acute postoperative pain trajectories, and prompt management of symptoms, are core determinants in postoperative care.

In our previous studies, latent curve models were used to identify the influential factors of postoperative pain trajectory in patients receiving intravenous and epidural patient-controlled analgesia (PCA) [25,26]. However, there is still a lack of studies which evaluate whether these factors are associated with the variations in acute pain trajectories over time after surgery for RCC. Therefore, we conducted this retrospective cohort study to explore influential factors of acute pain trajectories after RCC surgery using latent curve analysis. We hypothesized that patient demographics, surgical techniques, and the use of either intravenous patient-controlled analgesia (IVPCA) or patient-controlled epidural analgesia (PCEA) were of significant impact on the features of acute pain trajectories after RCC surgery, including the baseline pain level and rate of pain resolution. Clinical prediction models were further developed to provide more quantitative insights into the dynamic nature of postoperative pain trajectories and personalized pain management for RCC surgery.

## 2. Materials and Methods

### 2.1. Setting and Patient Selection

We conducted this retrospective cohort study in Taipei Veterans General Hospital after the approval of our Institutional Review Board (IRB-TPEVGH No. 2019-07-004BC), and written informed consent was waived. We reviewed the electronic medical records and collected data from patients undergoing curative surgery for RCC at this center from 2011 to 2018. Patients were excluded from the analysis if they had to receive postoperative ventilator support or intensive care beyond 24 h, were unable to report pain intensity, or had fewer than 2 postoperative pain assessments within 5 days after surgery.

### 2.2. Anesthesia and Analgesia Management

All patients received general anesthesia with propofol 1–2 mg·kg^−1^ and fentanyl 1–2 μg·kg^−1^ for induction. Rocuronium or cis-atracurium was used to facilitate endotracheal intubation and keep muscles relaxed during surgery. Anesthesia was maintained using sevoflurane 2–3 vol% or desflurane 6–8 vol% in a mixture of oxygen and air. For those receiving PCEA, combined epidural anesthesia was also employed intraoperatively with a combination of bupivacaine 0.25% and 5 μg·mL^−1^ fentanyl. An infusion pump (Gemstar™ Yellow, Hospira, Chicago, IL, USA) was used to deliver morphine 1 mg·mL^−1^ in normal saline, or a solution of bupivacaine 0.1% with 5 μg·mL^−1^ fentanyl for the IVPCA or PCEA users after surgery, respectively. PCA was typically used for 48 to 72 h after surgery, and patients were switched to oral acetaminophen or non-steroidal anti-inflammatory drugs thereafter. For the patients without IVPCA and PCEA, intravenous or intramuscular morphine or ketorolac was administered for postoperative pain control as necessary, and oral acetaminophen, non-steroidal anti-inflammatory drugs, and tramadol were used for pain relief whenever oral feeding was possible.

### 2.3. Pain Assessment and Data Acquisition

Postoperative pain was assessed at least once daily by a nurse in charge, with a self-report numeric rating scale (NRS) using an 11-point scale. Response options were arranged on a scale from “no pain” to “the worst pain”. The maximal daily NRS pain scores were retrieved during the first five postoperative days (POD) from the electronic medical databank, and served as the endpoints of the following latent curve analysis. All the patients were divided into three classes based on their analgesic choices (IVPCA, PCEA, and no PCA). The other collected covariates included age, sex, body weight and height, body mass index, American Society of Anesthesiologists (ASA) class, stage of cancer, diabetes, anesthesia time, perioperative transfusion, and surgical blood loss. All the medical records were extracted by a specialist anesthesiologist not involved in data analysis. Random samples of the collected data were thoroughly checked by the authors to ensure data quality.

### 2.4. Statistical Analysis

Patient attributes and maximal daily pain scores during the first five POD were expressed as mean ± SD or count with percentage. Comparisons of patient characteristics among the three PCA groups were performed using a one-way analysis of variance or a chi square test, as appropriate. Three types of latent curve models—basic, single predictor, and multiple predictor—were used to characterize the variations in pain scores over time, and to evaluate the effects of covariates on postoperative pain trajectories. The technical details of latent curve analysis refer to the previous literature [27,28]. In brief, the basic model was applied to estimate the baseline intercept and slope parameters without any covariate, while the single predictor model was used to evaluate the univariate effects of collected variables on the intercept and slope parameters. A backward model selection strategy with the exit criteria of *p* values greater than 0.05 was performed to identify independent predictors of the intercept and slope parameters and determine the final predictive model. The goodness of fit was assessed using a root mean square error of approximation (RMSEA) and a comparative fit index (CFI), and the values of RMSEA < 0.1 and CFI > 0.9 implied an acceptable fit to the data [29]. According to Hair et al., a minimum sample size of 300 is required to ensure a stable parameter estimation for structural equation modeling. This criterion was met in our study, because latent curve analysis is also a kind of structural equation modeling [30]. We have addressed this issue in the statistical analysis section. All latent curve analyses were implemented using AMOS 18.0 (SPSS Inc., Chicago, IL, USA). Other statistical analyses were conducted with the PASW 18.0 (SPSS Inc., Chicago, IL, USA).

## 3. Results

We included 861 patients with 3850 pain score observations in the analysis. Patient characteristics are presented in Table 1. The proportions of patients without PCA and with PCEA or IVPCA were 42%, 17.2%, and 40.8%, respectively. Note that there were significant differences in average maximal daily pain scores on the first three PODs among the three groups, and patients without PCA tended to have higher pain scores than those receiving PCEA and IVPCA (Table 1).

**Figure 1:**
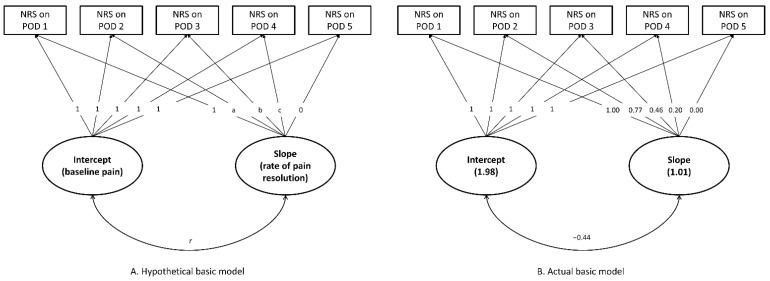
The basic latent curve model for pain trajectories after surgery for renal cell carcinoma. (**A**) Hypothetical basic model. (**B**) Actual basic model.

In the basic latent curve model, the estimated slope parameters (a, b, and c, Figure 1) from the POD 2 to POD 4 were 0.77, 0.46, and 0.20, respectively (all *p* < 0.001). The estimated intercept and slope parameters of the basic model were 1.98 and 1.01, respectively. Accordingly, the estimated daily mean pain score during the POD 1 and 5 can be calculated as follows:

POD 1: 1.98 + (1) × 1.01 = 2.99(1)

POD 2: 1.98 + (1.01) × 0.77 = 2.76, and so on.

**Figure 2:**
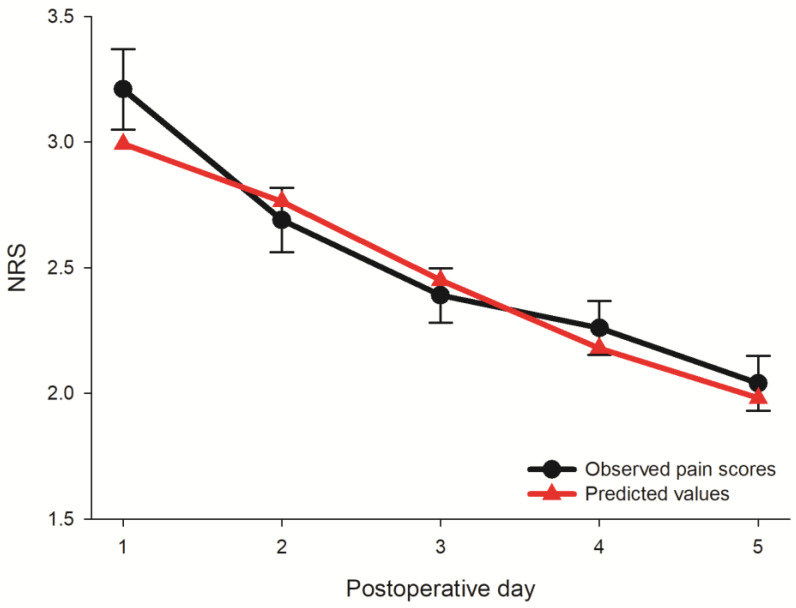
The observed maximal pain scores with their standard deviations and predicted values of the basic latent curve model after surgery for renal cell carcinoma.

Figure 2 presents the observed mean pain score with their standard deviation bars, and the predicted pain score from basic latent curve analysis for each POD. Notice that the largest difference between the observed and predicted pain scores was 0.22 units of NRS on the POD 1.

Table 2 shows the results of the single predictor latent curve analysis. Note that sex, ASA physical status, advanced cancer stage, surgical type and technique, and perioperative transfusion all had significant associations with the intercept parameters. Meanwhile, age, PCA use, and surgical technique were associated with the slope parameters. Other variables did not significantly affect either the intercept or slope parameters in the single predictor latent curve model.

**Figure 3:**
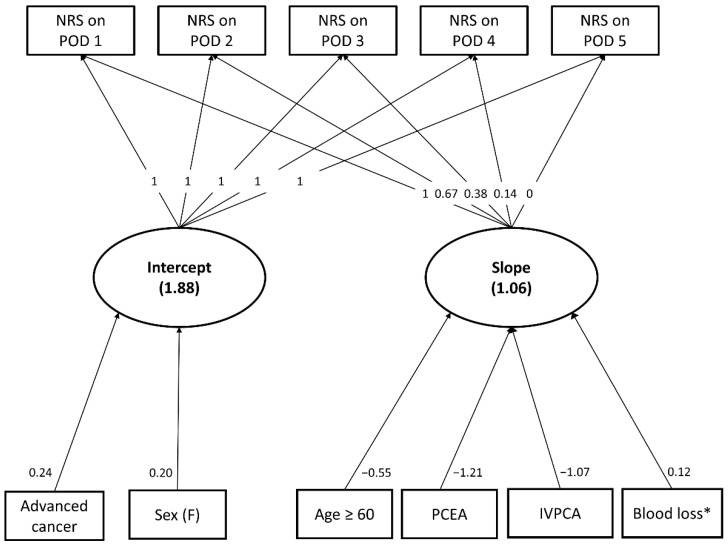
The final predictive model for pain trajectories after surgery for renal cell carcinoma.* On base-2 logarithmic scale.

Table 3 illustrates the final predictive latent curve model for pain trajectories after surgery for RCC. Two of the collected variables, advanced cancer stage, and female sex had significant effects on the intercept parameter of postoperative pain trajectories, and both factors were associated with higher baseline pain scores after surgery for RCC. Furthermore, age, PCA use, and surgical blood loss all had a significant influence on the slope parameters. Older patients tended to have faster pain resolution than their younger counterparts, while PCA users had a steeper declining pain trajectory than those without it. However, more surgical blood loss was associated with slower pain resolution after surgery for RCC. Note that laparoscopic or robotic surgery did not have any significant impact on the intercept or slope parameters after an adjustment for the other significant predictors of postoperative pain trajectories. The RMSEA and CFI values of the final model were 0.058 and 0.946, respectively, and the graphic presentation of the final predictive model is illustrated in Figure 3.

Based on the results, the predicted pain score during the first postoperative week can be estimated using the final predictive model. For example, the estimated maximal pain score on POD 2 can be calculated with the following formula:

[1.878 + 0.236 × (1 for cancer stage III or IV and 0 for stage I or II) + 0.204 × (1 for female and 0 for male)] + 0.670 × [1.061 – 0.548 × (1 for age ≥ 60 years and 0 for age < 60) – 1.214 × (1 for PCEA users and 0 for the others) – 1.068 × (1 for IVPCA users and 0 for the others) + 0.122 × (surgical blood loss on base 2 logarithmic scale)].(2)

## 4. Discussion

In the present study, we identified two factors associated with baseline pain, and three variables related to the rate of pain decrement, using latent curve analysis models in patients undergoing RCC surgery. We had also developed a model that predicts the trend in postoperative pain, although this model needed to be further validated before its use in clinical care. Female patients and those with an advanced stage of cancer would be more likely to have higher baseline pain scores. Of all the collected variables, older patients (older than 60), and PCA users tended to have faster pain relief, while patients with more surgical blood loss were associated with slower pain relief. Our analysis was based on a large number of patients to strengthen statistical power, which ensures more accurate and reliable results.

The extensive extraperitoneal incision, with or without rib resection, in the open nephrectomy usually induced severe pain. In the study of Kim et al., they performed open nephrectomy through a subcostal incision from the 12th rib to the suprapubic area [31]. The numeric pain score (NPS, ranging from 0 to 100) was used to evaluate the postoperative pain intensity, which showed that the median NPS at rest was 40 in patients receiving IVPCA at 24 h postoperative. Another study reported that the average pain intensity in patients was the NRS (0–10) 3.2 on day 1 after open nephrectomy [32]. In our study, the mean NRS pain score was 3.8 in the no-PCA group, 2.8 in the PCEA group and 2.7 in the IVPCA group, which was relatively lower than in the reports mentioned above. Of all the 861 patients included in our study, 399 (46.3%) patients received laparoscopic or robotic surgery, while 462 (53.7%) patients received open nephrectomy. Compared with open nephrectomy, some studies revealed that laparoscopic nephrectomy caused less pain and had significantly lower requirements in postoperative analgesics [33,34]. On the other hand, a prospective clinical trial showed that patients with RCC undergoing laparoscopic surgery had similar acute postoperative average pain scores to those undergoing open nephrectomy [35], which is consistent with our results. Our analysis demonstrated that neither laparoscopic nor robotic surgery had any significant impact on the baseline level or resolution rate of postoperative pain trajectories. Postoperative pain originating from laparoscopic or robotic surgery is possibly induced by pain at the inner surgical site, port sites, incision wound, and organ nociception [36], suggesting the importance of adequate pain control in these patients.

A few studies have examined the influential factors on acute postoperative pain trajectory in patients undergoing RCC surgery. Compared with isolated scores, trends in pain assessment scores over time are more helpful to improve pain research and optimize postoperative pain monitoring [12,25]. Our analysis showed that advanced cancer and female sex are two influential factors on the baseline pain level, while age≥ 60, usage of PCA, and more surgical blood loss are the other three influential factors on the rate of pain resolution. A prospective study, analyzing the risk factors of acute and chronic postoperative pain in 46 patients after nephrectomy, supposed that anxiety was the only preoperative factor that significantly correlated with more severe postoperative pain [32]. Further studies with validated designs, enough sample sizes, and powerful statistics are needed to identify the associated factors on postoperative pain trajectory in patients after RCC surgery.

With regard to the influence of aging on postoperative pain trajectory, previous studies had shown that older patients have either lower postoperative pain intensity or slightly slower pain relief from analgesics, compared with younger patients [13,14,37]. These findings were likely attributed to various changes with aging, including greater pain tolerance, overall decline in peripheral nociceptive function, and drug distribution in the human body [38,39]. Contrasting their results, our study demonstrated that advanced age (over 60 years old) was associated with faster postoperative pain relief, but did not affect the baseline pain level. These dichotomous findings were probably due to differences in patient characteristics, pain management, and analysis methods. Female patients had higher baseline pain scores after RCC surgery in this study. This result is in agreement with our previous studies and some previous reports [24,25,26,37]. A systemic review and meta-analysis showed that female sex was a predictor for poor postoperative pain control [37]. The sex difference may potentially be associated with complicated psychosocial and biological factors [37,40,41]. However, another large-scale cohort study demonstrated that a higher baseline postoperative pain score and faster pain relief were found in male patients [16].

For major surgery, the use of IVPCA and PCEA to effectively alleviate postoperative pain is well established. Furthermore, our study shows that patients using PCA are likely to have faster pain resolution. Recently, multimodal analgesia and the opiate-sparing technique, used within an enhanced recovery after surgery (ERAS) pathway, were advocated [42]. Further studies may be needed to evaluate the relationship between multimodal analgesia and the trends in postoperative pain trajectory. In addition, advanced cancer and more surgical blood loss were considered in our analysis as influential factors associated with more intense pain and slower pain resolution, respectively. Patients with advanced cancer may receive the surgery excision targeting the tumor and neighboring tissues, causing more extensive tissue injuries and intense postoperative pain. Concerning the factor of more surgical blood loss, a possible explanation is that intraoperative blood loss is generally used as a marker and predictor of operation and outcome [43], and more blood loss during the operation may be associated with the slower recovery of patients, thus causing slower postoperative pain resolution.

Our study has several limitations. First, this was a retrospective review of patients with RCC undergoing surgery at one institution. Patients were not randomized, and postoperative care was not standardized. Various unmeasured confounding factors such as preoperative pain, perioperative consumption of opioids, anxiety, and postoperative complications could not be further evaluated due to difficulties in data acquisition [14,20,24,41]. Secondly, instead of the dosage of analgesics consumption, we used NRS, a self-reporting subjective pain scale, as a tool to evaluate pain intensity. Although NRS had been considered a valid, reliable, and appropriate tool, a recent study showed different trajectories between pain intensity scores and opioid consumption [44]. This is a potential confounder of our study. Thirdly, postoperative comorbidities, such as impaired renal or liver function, were not included in the analysis, which may confound our results.

## 5. Conclusions

Our findings suggested that the factors of female sex, age ≥ 60, PCA use, advanced cancer, and surgical blood loss jointly influenced the postoperative pain trajectory in patients undergoing surgery for RCC. Applying this model to clinical practice may possibly facilitate personalized optimization of postoperative pain management based on individual characteristics after RCC surgery. Latent curve analysis provides insights into the dynamic features of the variations in pain trajectories and their determinants after surgery for RCC. Further studies may be needed to clarify the relationship between more variables, such as psychosocial factors and comorbidity of patients, and the postoperative pain trajectory in patients for RCC surgery.

## Figures and Tables

**Table 1:** Comparisons of patient characteristics and postoperative pain among the PCEA, IVPCA, and no PCA groups.

	No PCA (*n* = 362)	PCEA (*n* = 148)	IVPCA (*n* = 351)	*p*
Age ≥ 60 years	173 (47.8%)	64 (43.2%)	178 (50.7%)	0.306
Sex, female	112 (30.9%)	46 (31.1%)	117 (33.3%)	0.767
Body height, cm	165 ± 9	166 ± 8	164 ± 9	0.040
Body weight, kg	69.8 ± 14.0	70.2 ± 12.6	69.0 ± 13.5	0.591
Body mass index, kg·m^−2^	25.6 ± 4.0	25.4 ± 3.9	25.7 ± 4.1	0.840
ASA class ≥ 3	111 (30.7%)	39 (26.4%)	97 (27.6%)	0.529
Charlson comorbidity index	4.1 ± 1.8	3.8 ± 1.8	4.0 ± 1.7	0.208
Advanced cancer (stage III, IV)	97 (26.8%)	45 (30.4%)	119 (33.9%)	0.119
Tumor side				0.839
Left	172 (47.5%)	64 (43.2%)	169 (48.1%)	
Right	183 (50.6%)	80 (54.1%)	173 (49.3%)	
Bilateral	7 (1.9%)	4 (2.7%)	9 (2.6%)	
Anesthesia time, min *	8.5 ± 0.4	8.5 ± 0.5	8.5 ± 0.5	0.172
Surgical type				0.330
Radical nephrectomy	230 (63.5%)	87 (58.8%)	205 (58.4%)	
Partial nephrectomy	132 (36.5%)	61 (41.2%)	146 (41.6%)	
Surgical technique				<0.001
Open	109 (30.1%)	134 (90.5%)	210 (59.8%)	
Laparoscopic	105 (29.0%)	3 (2.0%)	65 (18.5%)	
Robotic	148 (40.9%)	11 (7.4%)	76 (21.7%)	
Surgical approach **				<0.001
Transperitoneal	235 (64.9%)	14 (9.4%)	138 (39.3%)	
Retroperitoneal	18 (5.0%)	0 (0.0%)	3 (0.9%)	
Surgical drain				0.264
0	19 (5.2%)	10 (6.8%)	15 (4.3%)	
1	332 (91.7%)	128 (86.5%)	322 (91.7%)	
≥2	11 (3.0%)	10 (6.8%)	14 (4.0%)	
Surgical blood loss, mL *	7.4 ± 1.9	8.0 ± 2.0	7.8 ± 1.9	<0.001
Perioperative transfusion	69 (19.1%)	42 (28.4%)	95 (27.1%)	0.106
Mean NRS pain score				
POD 1	3.8 ± 2.	2.8 ± 2.0	2.7 ± 2.0	<0.001
POD 2	3.1 ± 2.1	2.2 ± 1.3	2.5 ± 1.7	<0.001
POD 3	2.6 ± 1.7	2.2 ± 1.4	2.3 ± 1.4	0.012
POD 4	2.3 ± 1.6	2.1 ± 1.4	2.3 ± 1.6	0.553
POD 5	2.0 ± 1.5	2.0 ± 1.3	2.1 ± 1.4	0.920

Values are mean ± standard deviation or counts (percent). ASA: American Society of Anesthesiologists; NRS: numeric rating scale; PCA: patient-controlled analgesia; PCEA: patient-controlled epidural analgesia; IVPCA: intravenous patient-controlled analgesia; POD: postoperative day. * On base-2 logarithmic scale. ** Surgical approach for laparoscopic or robotic surgery.

**Table 2:** Effects of collected variables on the intercept and slope parameters in the single predictor model.

	Intercept			Slope		
	Estimate	SE	*p*	Estimate	SE	*p*
Age ≥ 60	0.151	0.093	0.107	−0.56	0.156	<0.001
Sex (female vs. male)	0.204	0.1	0.04	−0.162	0.165	0.326
Body height	−0.004	0.005	0.468	0.004	0.009	0.633
Body weight	−0.002	0.003	0.584	0.002	0.006	0.679
Body mass index	−0.003	0.012	0.821	0.002	0.019	0.924
ASA class (≥ 3 vs. < 3)	0.211	0.103	0.04	−0.052	0.17	0.758
Charlson comorbidity index	0.002	0.026	0.953	−0.055	0.044	0.210
Cancer (stage III, IV vs. I, II)	0.243	0.101	0.016	−0.145	0.167	0.386
PCA use						
PCEA vs. nil	−0.006	0.134	0.962	−1.081	0.22	<0.001
IVPCA vs. nil	0.122	0.104	0.238	−1.065	0.17	<0.001
Tumor side (left vs. right)	−0.036	0.094	0.699	−0.133	0.156	0.395
Anesthesia time *	0.083	0.102	0.416	0.116	0.169	0.493
Surgical type (radical vs. partial)	0.238	0.095	0.012	−0.113	0.157	0.475
Surgical technique						
Laparoscopic vs. open	0.081	0.122	0.504	0.623	0.202	0.002
Robot vs. open	−0.248	0.109	0.023	0.154	0.181	0.394
Surgical drain	−0.079	0.120	0.511	0.074	0.198	0.711
Surgical blood loss *	0.027	0.024	0.27	0.055	0.041	0.172
Perioperative transfusion	0.282	0.108	0.009	0.012	0.18	0.949

SE: standard error. ASA: American Society of Anesthesiologists. * On base-2 logarithmic scale.

**Table 3:** The multiple predictor latent curve model after backward model selection.

	Estimate	SE	*p*
Intercept			
Sex (female vs. male)	0.236	0.094	0.028
Cancer (stage III, IV vs. I, II)	0.204	0.093	0.012
Slope			
Age ≥ 60	−1.214	0.207	<0.001
PCA use			
PCEA vs. nil	−1.068	0.159	<0.001
IVPCA vs. nil	0.122	0.038	<0.001
Surgical blood loss *	0.068	0.013	0.001

SE: standard error. * On base-2 logarithmic scale.

## Data Availability

All data are available from the author directly.
